# Supplementary material for: EMG-assessed paratonia: A novel approach to investigating motor response inhibition in healthy subjects
Source: PLoS One. 2024 Dec 18;19(12):e0315274. doi: 10.1371/journal.pone.0315274 (PMC11654976; doi:10.1371/journal.pone.0315274)
Supplement: Attachment — Submitted filename: Reviewer 1 and 2.docx [file pone.0315274.s002.docx]

***Reviewer #1:***

*The article introduces an innovative approach to assessing motor response inhibition through EMG-assessed paratonia in healthy individuals. This approach represents a valuable contribution to the existing body of literature on motor inhibition, expanding beyond traditional methods such as the Go/No-Go and Stop Signal Tasks.*

*The study’s results reveal a significant relationship between physical activity levels and motor inhibition, enhancing its relevance for both neuroscience research and sports science.*

*While the manuscript is well-structured and the methodology appears robust, there are a few areas that would benefit from further clarification and refinement.*

**Authors' Response:**

We sincerely thank the reviewer for the time and effort dedicated to reviewing our manuscript, as well as for the valuable suggestions that have contributed to its improvement. We have enthusiastically addressed each comment and request, making the suggested modifications. All changes have been implemented and highlighted in yellow in the revised manuscript to facilitate the review.

Introduction:

***Reviewer #1:***

*The introduction mentions that physical activity has an impact on motor inhibition, but this connection could be explained in more detail.* *Adding a brief explanation of how physical activity affects the brain circuits involved in motor inhibition (e.g. through training executive functions or improving motor control) could provide a stronger context for the study's hypothesis.*

**Authors' Response:**

Thanks for the suggestion. In the revision of the manuscript, specific information was added on how physical activity can have an impact on motor inhibition:

“This effect may be due to the way in which physical activity, particularly in dynamic and unpredictable environments, enhances executive function and motor control, both of which are critical for motor response inhibition”

2. Methodology:

***Reviewer #1:***

*It would be helpful to provide more detail on how self-reported physical activity levels (METs) were verified. Were there any objective measures (e.g., wearable devices) to cross-check these self-reports?*

**Authors' Response:**

The physical activity levels were only based on the participant's self-report without any objective verification to ensure their veracity.

***Reviewer #1:***

*While the methodology is robust, additional clarity on the rationale for the chosen movement speeds (40 BPM and 100 BPM) would strengthen the manuscript. Why were these specific speeds selected, and how do they relate to real-world movements in the context of sports or daily activities?*

**Authors' Response:**

In a recent study (Marinelli et al., 2022) conducted at speeds of 60 and 100 BPM, we found that paratonia is influenced by movement velocity; however, this characteristic was not evident under all test conditions, as only facilitatory paratonia of the biceps and oppositional paratonia of the triceps increased with speed. We hypothesized that comparing movements at lower speeds, such as 40 BPM, and higher speeds, like 120 BPM, might better highlight the velocity dependence of paratonia across all test conditions. Therefore, in this study, we selected a lower speed of 40 BPM, compared to the previous 60 BPM, to test this hypothesis. Higher speeds, such as 120 BPM, which would better simulate conditions used in sports contexts, were not chosen to avoid passive movements at such velocities potentially eliciting phasic reflexes, which could interfere with and obscure paratonic activity.

With the revised manuscript we include additional information in order to justify the passive movement speeds used in the study:

“The 40 BPM cadence was selected to simulate slow and precise movements characteristic of certain sports activities. Conversely, the 100 BPM cadence was chosen to represent rapid and explosive movements typical of other sports contexts. We deliberately avoided using higher movement speeds to prevent the elicitation of phasic reflexes, which could interfere with and obscure the assessment of paratonic activity”.

***Reviewer #1:***

*What is the scientific rationale behind the decision to interpolate the data to 101 points for each flexion and extension, instead of using a more conventional number such as 100?*

**Authors' Response:**

Interpolating data to 101 points, rather than 100, is a common practice in time-normalization of movement cycles, such as flexion and extension phases. This approach ensures that both the start (0%) and end (100%) of the movement are included, providing a complete representation of the cycle. By dividing the movement into 100 equal segments, 101 data points are generated, capturing every critical phase, including the exact endpoints. This method enhances the accuracy and consistency of analyses across different trials and subjects. For instance, in MATLAB's *interp1* function, when downsampling data, it's recommended to use 101 points to divide the data into 100 equal segments, ensuring that all key points are represented.

***Reviewer #1:***

*It might be useful to provide more details on the procedure used to perform the maximum voluntary contraction, which was employed for the normalization of the data.*

**Authors' Response:**

We did an oversight by not specifying that the contraction was an isometric contraction. So an important aspect has been added in the revised study

***Reviewer #1:***

It would be helpful to provide a reference for the methodology used to process the raw EMG signal, specifically the band-pass filtering in the 20-450 Hz range, followed by rectification and smoothing using a 4th order Butterworth low-pass filter with a 5 Hz cut-off frequency.

**Authors' Response:**

The reference has been added as kindly requested: “Cotellessa F, Bragazzi NL, Trompetto C, Marinelli L, Mori L, Faelli E, et al. Improvement of motor task performance: Effects of verbal encouragement and music—Key results from a randomized crossover study with electromyographic data. Sports. 2024;12(8):210. doi:10.3390/sports12080210.”

***Reviewer #1:***

Similar to before, it would be useful to include a reference to the methodology used to calculate the area under the EMG signal curve using the trapezoidal method, particularly for calculating the area during flexion and extension periods.

**Authors' Response:**

The reference has been added as kindly requested: “Yeh S-T. Using trapezoidal rule for the area under a curve calculation. In: Proceedings of the 27th Annual SAS Users Group International Conference; 2002 Apr 14–17; Orlando, FL. Cary (NC): SAS Institute Inc.; 2002. Paper 229-27.”

***Reviewer #1:***

*Statistical analysis: The statistical analysis reads fine, but further clarification is recommended. How the sampling was conducted is unclear. Can the authors specify how they recruited the participants (randomly, via snowballing, purposively sampling, convenience sampling, etc.)?*

*Do they consider the participants’ fndings generalizable to the entire population? Can the authors reflect and comment on these aspects?*

**Authors' Response:**

Participants were recruited using a purposive sampling approach to ensure that our sample included individuals with different levels of physical activity. Specifically, we recruited sedentary individuals from corporate, academic, and other non-athletic environments, amateur athletes through local sports clubs, and professional athletes from established sports teams. This approach was chosen to allow for a clear comparison across groups representing different activity levels.

Regarding the generalizability of our findings, our study focused on a specific subset of the population (healthy individuals under the age of 30). Therefore, while our findings are relevant within this demographic, they may not be directly generalizable to other age groups or to the broader population, particularly those with health conditions or varying age demographics.

***Reviewer #1:***

*3. Results: The presentation of the results is solid and well structured. Furthermore, the presentation of the results through table and figures is well realized, allowing a clear visualization of the main findings.*

**Authors' Response:**

Thank you for your positive feedback

***Reviewer #1:***

*4. Discussion:*

*The difference between ‘open-skill’ and ‘closed-skill’ sports (e.g. swimming and tennis) is discussed, but a clear explanation of why athletes of ‘open-skill’ sports should have more efficient circuits is lacking? This could be related to the neuroplasticity induced by specific physical training, but the explanation is too superficial.*

**Authors' Response:**

A more thorough explanation of why athletes in ‘open-skill’ sports more efficient circuits should have been added in the revised manuscript: “Previous studies have shown that the ability to inhibit unwanted motor activity is more efficient in professional athletes involved in open-skill sports than in those involved in closed-skill sports. This finding has been attributed to the fact that open-skill athletes must constantly adapt to changing situations influenced by the actions of other players and the environment, requiring a heightened capacity for motor control and inhibition of automatic responses. This constant adaptation is likely to enhance their ability to quickly inhibit automatic or pre-planned responses, allowing for greater flexibility and precision in real-time decision making. In contrast, athletes in closed-skill sports, who train in more stable and predictable conditions, tend to rely less on these inhibitory control processes because their performance relies more on repetition and consistency rather than the ability to adapt quickly to external changes [32,33].”

***Reviewer #1:***

*5. Minor Issues:*

*There are a few minor grammatical errors throughout the manuscript (e.g., missing articles). A careful proofreading would address these.*

**Authors' Response:**

We thoroughly proofread the manuscript and corrected these issues, including missing articles and other minor mistakes

***Reviewer #1:***

*Overall, the manuscript is of high quality, and the study provides meaningful contributions to the understanding of motor response inhibition. After addressing the minor revisions, I believe the manuscript will be suitable for publication.*

**Authors' Response:**

Thank you for your positive feedback

***Reviewer #2***

*The aim of the present manuscript was to investigate whether EMG-assessed paratonia could be used to test motor inhibition also in cognitively unimpaired individuals, other than in patients with Alzheimer Disease as previously demonstrated. Secondly, it was aimed to test whether the investigation of EMG-assessed paratonia in healthy subjects is able to reveal a well-known feature of motor response inhibition, specifically its correlation with the level of physical activity. The paper is well written; the study methodology is correct and the results are adequately reoprted, demostrating the efficacy of this neurophysiological approach not only in patients with dementia but also in healthy subjects without clinical manifestation of paratonia. I have no comment*

**Authors' Response:**

Thank you, Reviewer #2, for your positive and thorough evaluation of our manuscript. We are glad to hear that you found the study well-written and the methodology sound, as well as that the results were adequately reported. Your feedback reassures us of the robustness of our neurophysiological approach and its broader applicability. Thank you again for your review
